# Psychological Resilience, Symptom Burden, and Supportive Care Needs in Chemotherapy Patients: A Path Analysis

**DOI:** 10.1002/brb3.70851

**Published:** 2025-11-21

**Authors:** Rukiye Burucu, Işın Cantekin

**Affiliations:** ^1^ Rukiye Burucu, Nursing Department Necmettin Erbakan University Kamil Akkanat Faculty of Health Sciences Konya Turkey; ^2^ Işın Cantekin, Nursing Department Necmettin Erbakan University Kamil Akkanat Faculty of Health Sciences Konya Turkey

**Keywords:** Cancer, chemotherapy, psychological resilience, supportive care, symptoms

## Abstract

**Introduction and Aim:**

While chemotherapy contributes to the survival of cancer patients, it also leads to various psychological and physiological problems, increasing the need for supportive care. This study aimed to determine the relationship between psychological resilience, chemotherapy symptoms, and supportive care needs in patients undergoing chemotherapy.

**Method:**

This is a descriptive and correlational study. Data were collected using the Descriptive Characteristics Form, the Psychological Resilience Scale (PRS), the Nightingale Symptom Assessment Scale (N‐SAS), and the Supportive Care Needs Scale‐Short Form (SCNS‐SF). Data collection was conducted face‐to‐face in the chemotherapy unit of a university hospital. Analyses were performed using the Statistical Package for Social Sciences (SPSS) 29.0 and Hayes Process Macro V3.4.

**Results:**

A total of 113 patients participated in the study. The mean age of the participants was 56.91 years (SD = 10.917), the mean PRS score was 106.59 (SD = 5.194), the mean N‐SAS score was 2.14 (SD = 0.423), and the mean SCNS‐SF score was 72.58 (SD = 9.250). The effect of PRS on N‐SAS was primarily direct and statistically significant (*β* = −0.019; *p* = 0.011). However, the effect of PDÖ on SCNS‐SF was not statistically significant (*β* = 0.017; *p* = 0.752). Participants generally demonstrated high levels of psychological resilience, a low impact of symptoms on quality of life, and moderate levels of supportive care needs.

**Conclusion:**

The psychological resilience levels of the patients were generally high. The impact of symptoms on patients' quality of life was low. While an increase in symptoms negatively affected psychological resilience, the effect of psychological resilience on supportive care needs was limited and indirect.

## Graphical abstract

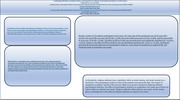

## 1 Introduction

One of the methods used in cancer treatment is chemotherapy. Chemotherapy administered over a prolonged period contributes to extending the patient's lifespan (Tasnim et al. 2020). However, it may also lead to various psychological problems such as anxiety, depression, anger, insomnia, and emotional fluctuations. High psychological resilience, on the other hand, contributes to more positive coping with these issues. Psychological resilience is defined as an individual's ability to adapt to stressful events and manage the situation effectively (Ayhan et al. 2021). Several factors affect the psychological resilience of patients undergoing chemotherapy (Ölmez and Karadağ 2022), and chemotherapy itself is one of these factors (Coolbrandt et al. 2018).

Chemotherapy can negatively affect quality of life due to the psychological, gastrointestinal, and nutritional symptoms it causes (Harris et al. 2022). In individuals diagnosed with cancer, poor management of symptoms can adversely affect their overall health (Ioannou et al. 2022). Symptom management is a vital component of nursing care (Prip et al. 2018), and it has been shown that nursing interventions significantly reduce symptom severity (Kwok et al. 2022).

In individuals receiving chemotherapy, an increase in symptoms (Hammersen et al. 2023), unresolved needs, and unaddressed demands can lead to an increase in supportive care needs (Sender et al. 2020). Supportive care is a patient‐centered approach that assists patients diagnosed with cancer and their families in coping with the illness throughout the entire course of the disease, beginning at diagnosis (Steele and Fitch 2008). A person's supportive care needs may include emotional, spiritual, health‐related, social, cognitive, and physical dimensions. Identifying these needs and producing solutions is one of the key responsibilities of nurses (Tel Aydın and Günay 2020).

In order for individuals undergoing chemotherapy to manage the process effectively, they need to adapt to their condition. This situation can be explained by the Roy Adaptation Model. This model allows for the assessment of patients’ adaptation in various domains such as physical, psychological, social, and role function, and facilitates the development of interventions based on individual needs (Abdallah Abdel‐Mordy et al. 2021). Therefore, it would be theoretically appropriate to base this study on the Roy Adaptation Model.

In the literature, various aspects have been studied separately, such as the impact of chemotherapy on mental health (Zhao et al. 2024), chemotherapy and symptom management (Cetin et al. 2022, Bellas et al. 2022), and supportive care needs in patients receiving chemotherapy (Bellas et al. 2022, Chien et al. 2022). However, no study has been found that examines the relationship between psychological resilience, chemotherapy‐related symptoms, and supportive care needs in patients receiving chemotherapy. Therefore, the aim of this study is to determine the relationship between psychological resilience, chemotherapy symptoms, and supportive care needs in patients undergoing chemotherapy through path analysis. The findings of this study are expected to contribute to the provision of supportive care tailored to the psychological resilience and symptom status of patients receiving chemotherapy.

## 2 Research Questions

1. Do patients have a high level of psychological resilience?
2. Are patients highly affected by problems arising from the disease or its treatment?
3. Do patients have extensive supportive care needs?
4. What is the relationship between psychological resilience, symptom distress, and supportive care needs?

## 3 Methods

This is a descriptive and correlational study.

### 3.1 Dependent Variables

Psychological resilience, level of symptom impact, and supportive care needs.

### 3.2 Independent Variables

Socio‐demographic variables (gender, age, marital status, parental status, education level, and income level), type of cancer, duration of illness, frequency of chemotherapy, and route of chemotherapy administration.

**FIGURE 1:**
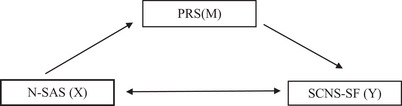
The conceptual model of the study.

According to the literature, symptom management in patients undergoing chemotherapy can positively influence psychological resilience (Arefian and Asgari‐Mobarakeh 2024, Hu et al. 2024). In the same patient group, it has also been emphasized that as psychological resilience increases, supportive care needs tend to decrease (Soyer Er and Erkan 2023). Moreover, a strong relationship between symptom burden and supportive care needs in individuals with cancer has been identified (Miranda et al. 2025, Erdoğan Yüce et al. 2021). Based on this information, the model presented in Figure 1 was developed.

### 3.3 Population and Sample

**FIGURE 2:**
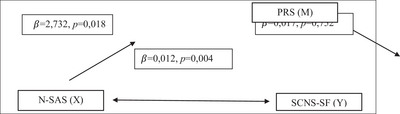
Structural Equation Modeling analysis result.

The study was conducted in the Chemotherapy Unit of Necmettin Erbakan Medical Faculty of Medicine Hospital. The population consisted of all patients receiving treatment in the Chemotherapy Unit of XXX Faculty of Medicine Hospital. Three different scales were used in the study. Sample size was calculated separately based on each dependent variable, and the largest required size was accepted as the final sample size. A previous study revealed that various factors affected the psychological resilience of patients diagnosed with prostate cancer. That study included seven variables and applied regression analysis (*R* ^2^ = 0.11) (Chien et al. 2022). According to this result, a power analysis was conducted using the G*Power 3.1.9.4 program. With an effect size of 0.124, power of 0.85, and an error margin of 0.05, it was determined that a minimum of 92 participants was required. In this study, data were collected from 113 participants, and the post hoc power was calculated as 0.91. Figure 2

#### 3.3.1 Inclusion Criteria

Age > 18 years, having received at least three cycles of chemotherapy or radiotherapy, and no communication difficulties.

#### 3.3.2 Exclusion Criteria

Refusal to participate in the study, being in the terminal phase, or receiving psychiatric treatment due to mental health issues.

### 3.4 Data Collection Forms

Data were collected using the Descriptive Characteristics Form, Psychological Resilience Scale (PRS), Nightingale Symptom Assessment Scale (N‐SAS), and the Short Form of the Supportive Care Needs Survey.

### 3.5 Descriptive Characteristics Form

This form was prepared by the researcher based on the literature (Ölmez and Karadağ 2022, Kwok et al. 2022, Hammersen et al. 2023, Soyer Er and Erkan 2023, Pekmezci Barut et al. 2022). It consists of 12 questions covering sociodemographic information and disease characteristics.

### 3.6 Psychological Resilience Scale

The scale was developed by Friborg et al. (2003) to measure individuals' psychological resilience. Originally five‐dimensional, it was revised into six dimensions in 2005: self‐perception (items: 1, 7, 13, 19, 28, and 31), future perception (2, 8, 14, and 20), structured style (3, 9, 15, and 21), social competence (4, 10, 16, 22, 25, and 29), family cohesion (5, 11, 17, 23, 26, and 32), Social resources (6, 12, 18, 24, 27, 30, and 33). It uses a 5‐point Likert scale, with response interpretations varying by item. In this study, a higher score indicates higher psychological resilience. The following items are reverse‐scored: 1, 3, 4, 8, 11, 12, 13, 14, 15, 16, 23, 24, 25, 27, 31, and 33. For reverse‐scoring, responses are subtracted from 6. Total scores range from 33 to 165; midpoints are 2.5 and 99. Higher scores indicate greater psychological resilience. Cronbach's alpha for the subscales ranges from 0.75 to 0.86 (Basım and Çetin 2011); in this study, it was 0723.

### 3.7 Nightingale Symptom Assessment Scale

Developed by Can and Aydıner in 2009, this scale measures quality of life in cancer patients. It contains 38 items in three subscales: physical well‐being (items: 1–4, 6–15, 23–27, and 37), social well‐being (items: 5, 16–22), and psychological well‐being (items: 28–36, 38). Each item reflects the degree of impact from disease/treatment‐related problems. The scale uses a 5‐point Likert‐type scoring: “No” (0), “Very little” (1), “Some” (2), “Quite a bit” (3), and “Very much” (4). Subscale scores are calculated as total item scores divided by the number of items. Total scale score is the average of the three subscale means. Higher scores indicate worse well‐being and greater symptom impact. Cut‐off points may be applied if needed; 0–0.50: Very good, 0.51–1.50: Good, 1.51–2.50: Moderate, 2.51–3.50: Poor, 3.51–4.00: Very poor. Cronbach's alpha is 0.81–0.87 for subscales and 0.93 overall (25). In this study, it was 0830.

### 3.8 Short Form of the Supportive Care Needs Survey (SCNS‐SF)

Developed by McElduff, Boyes, Zucca, and Girgis (2004), this scale identifies and assesses the needs of cancer patients. It includes 31 items across five subscales: psychological needs (items: 1–9), health system and information (Sender et al. 2020, Steele and Fitch 2008, Tel Aydın and Günay 2020, Abdallah Abdel‐Mordy et al. 2021, Zhao et al. 2024, Cetin et al. 2022, Bellas et al. 2022, Chien et al. 2022, Arefian and Asgari‐Mobarakeh 2024, Hu et al. 2024), physical and daily living (Soyer Er and Erkan 2023, Miranda et al. 2025, Erdoğan Yüce et al. 2021, Pekmezci Barut et al. 2022, Basım and Çetin 2011), patient care and support (Can and Aydiner 2011, Aksuoğlu and Şenturan 2016, George and Mallery 2021, Brace et al. 2016), and sexuality (Hayes 2022, Baeda and Nurwahyuni 2022, Xu et al. 2023). It uses a 5‐point Likert scale (1 = no need at all, 2 = no need, 3 = low need, 4 = some need, 5 = high need). Scores are standardized using the formula: (Total score × 100)/(*m* × [*k* −1]), where *m* is the number of items in the subscale and *k* is the number of Likert scale options. Total scores range from 31 to 155; higher scores indicate higher supportive care needs. Cronbach's alpha ranges from 0.93 to 0.96 for subscales and is 0.93 overall (Aksuoğlu and Şenturan 2016); in this study, it was 0.92.

### 3.9 Data Collection Procedure

Data were collected face‐to‐face by the researcher in the chemotherapy unit. Patients were approached upon their arrival at the unit, and data collection forms were administered immediately before the initiation of chemotherapy. This approach aimed to ensure that patients could provide accurate responses prior to experiencing any medication‐related side effects. The interviews were conducted in a private consultation room, where only the patient and the researcher were present, thus maintaining confidentiality.

### 3.10 Ethical Considerations

Ethical approval for this study was granted by the Scientific Research Ethics Committee of the Faculty of Health Sciences at Necmettin Erbakan University, and institutional permission was obtained from XXX Faculty of Medicine Hospital. The research was conducted in full compliance with the principles outlined in the Declaration of Helsinki. Throughout all phases of reporting, the STROBE checklist was rigorously applied to ensure transparency and quality. Authorization for the use of the scales was secured via email from the original authors, who established their Turkish validity and reliability. Informed consent, both written and verbal, was obtained from all participants prior to data collection.

### 3.11 Statistical Analyses

Data were analyzed using the Statistical Package for Social Sciences (SPSS) 29.0. Continuous variables are presented as means (x̄) and standard deviations (SD); categorical variables are presented as numbers (*n*) and percentages (%). Normality was assessed via skewness and kurtosis values (between –1 and +1) (George and Mallery 2021), bell curve inspection, normal *Q*–*Q* plots, and comparison of mean, median, and mode values. Since most continuous variables met the criteria for normal distribution, parametric tests were used—for two‐group comparisons: independent samples *t*‐test, for comparisons with three or more groups: one‐way ANOVA; if variance homogeneity was not met: Welch's ANOVA; for post hoc tests: Tamhane's T2 was used. Pearson correlation was used to evaluate relationships between continuous variables. Correlation interpretation (*r* = 0.1–0.29: weak, *r* = 0.3–0.69: moderate, *r* = 0.7–0.99: strong) (Brace et al. 2016). Mediation analyses were conducted using Hayes' PROCESS Macro V3.4 (Hayes 2022). A significance level of *p* < 0.05 was accepted for all statistical tests.

The mean age of the 113 participants included in the study was 56.91 (SD = 10.917). The mean total score of the PRS was 106.59 (SD = 5194), the mean score of the N‐SAS was 2.14 (SD = 0423), and the mean total score of the Supportive Care Needs Survey—Short Form (SCNS‐SF) was 72.58 (SD = 9250). Most of the participants were male (54.0%; *n* = 61), married (91.2%; *n* = 103), had primary education (59.3%; *n* = 67), were unemployed (81.4%; *n* = 92), and lived with their spouses (86.7%; *n* = 98). Clinically, most participants had a chronic illness in addition to cancer (39.8%; *n* = 45), were diagnosed with gastrointestinal cancer (25.7%; *n* = 29), had been diagnosed for over 24 months (38.9%; *n* = 44), were receiving chemotherapy beyond the 3rd cycle (79.6%; *n* = 90), and had also received radiotherapy as a treatment (37.2%; *n* = 42) (Table 1).

**TABLE 1:** Distribution of the patients’ sociodemographic characteristics (*n* = 113).

Variable	Category	$\bar{x}$	SD
Age		56.91	10.917
PRS total		106.59	5.194
N‐SAS total		2.14	0.423
SCNS‐SF total		72.58	9.250
		***N***	**%**
Gender	Male	61	54.0
	Female	52	46.0
Marital status	Married	103	91.2
	Single	10	8.8
Educational status	Illiterate	9	8.0
	Primary education	67	59.3
	Secondary education	28	24.8
	University and above	9	8.0
Employment status	Employed	21	18.6
	Unemployed	92	81.4
Person living with	Spouse	98	86.7
	Children	8	7.1
	Alone	7	6.2
Chronic illness other than cancer	One chronic disease	45	39.8
	Two chronic diseases	38	33.6
	More than three chronic diseases	30	26.5
Type of cancer	Breast	25	22.1
	GIS	29	25.7
	Lung	20	17.7
	Hematologic	12	10.6
	Other	27	23.9
Time since cancer diagnosis	6 months or less	25	22.1
	7–12 months	16	14.2
	13–18 months	15	13.3
	19–24 months	13	11.5
	> 24 months	44	38.9
Chemotherapy cycle number	3rd cycle	23	20.4
	> 3 cycles	90	79.6
Other treatment type received	No other treatment	31	27.4
	Radiotherapy	42	37.2
	Surgery	40	35.4

This section examines the mean scores from the PRS, N‐SAS, and SCNS‐SF scales based on various sociodemographic and clinical characteristics, as well as the statistical significance of differences between these scores. Significant differences were observed across all three scales based on education level. Participants with a university degree or higher had the highest PRS mean score ($\bar{x}=$111.11; SD = 3756), while illiterate participants had the highest mean scores for N‐SAS ($\bar{x}=$2.74; SD = 0681) and SCNS‐SF ($\bar{x}=$84.00; SD = 7018). These differences were statistically significant (*p* < 0.05). In terms of chronic illnesses, the group with only one chronic disease had the highest SCNS‐SF score ($\bar{x}=$75.69; SD = 8463), while those with three or more chronic diseases had the lowest ($\bar{x}=$69.37; SD = 8616). For cancer type, only the N‐SAS scores showed a significant difference. The highest N‐SAS scores were among those with hematologic cancers ($\bar{x}=$2.33; SD = 0119), and the lowest were among gastrointestinal cancer patients ($\bar{x}=$1.91; SD = 0316). Based on the time since diagnosis, significant differences were observed across all three scales. The highest PRS scores were among those diagnosed 19–24 months ago ($\bar{x}=$108.62; SD = 5501), while the highest N‐SAS scores were seen in the 7–12 months group ($\bar{x}=$2.41; SD = 0639), and the highest SCNS‐SF scores were again in the 19–24 months group ($\bar{x}=$77.77; SD = 9993). Significant differences in PRS scores were also found based on the chemotherapy cycle, with the highest average found in patients in the 3rd cycle ($\bar{x}=$108.87; SD = 4865). No significant differences were found for other variables (*p* > 0.05). Table 2

**TABLE 2:** Distribution of participants’ scale scores by independent variables (*n* = 113).

Variable	Category	PRS		N‐SAS		SCNS‐SF	
		$\bar{x}$	SD	$\bar{x}$	SD	$\bar{x}$	SD
Gender	Male	105.95	4.818	2.16	0.471	72.74	10.147
	Female	107.35	5.555	2.13	0.362	72.40	8.168
		*t*: 1.187	***p***: 0.156*	*t*: 1.098	***p***: 0.297*	*t*: 5.369	***p***: 0.849*
Marital status	Married	106.51	5049	2.16	0414	72.34	9316
	Single	107.40	6786	1.95	0480	75.10	8569
		*t*: 2.456	***p*** : 0.609*	*t*: 0.223	***p***: 0.126*	*t*: 0.869	***p***: 0.370*
Educational status	Illiterate^a^	103.00	5220	2.74	0681	84.00	7018
	Primary education^b^	107.16	4801	2.08	0352	69.24	8846
	Secondary education^c^	104.93	5340	2.24	0286	75.64	6993
	University and above^d^	111.11	3756	1.74	0299	76.56	6876
		*t*: 5.534 d > *b* > c > a	*p*: 0.001**	*t*: 12.785 a > c > b > d	*p*: 0.000**	*t*: 11.649 a > d > c > b	*p*: 0.000**
Employment status	Employed	106.43	4925	2.10	0201	72.67	9156
	Unemployed	106.63	5278	2.15	0459	72.57	9321
		*t*: 0.289	***p***: 0.873*	*t*: 13.781	***p***: 0.570*	*t*: 0.143	***p***: 0.964*
Person living with	Spouse	106.34	5199	2.15	0395	71.90	9291
	Children	109.38	3204	2.24	0509	77.75	8779
	Alone	107.00	6557	1.99	0682	76.29	7251
		*t*: 1.295	***p***: 0.278**	***t***: **0.708**	***p***: 0.495**	*t*: 2.119	***p***: 0.125**
Chronic illness other than cancer	One chronic disease^a^	106.84	5448	2.17	0505	75.69	8463
	Two chronic diseases^b^	106.24	4487	2.17	0332	71.45	9706
	More than three chronic diseases^c^	106.67	5762	2.07	0394	69.37	8616
		*t*: 0.143	***p***: 0.867**	*t*: 0.551	***p***: 0.578**	*t*: 4.965 a > b > c	***p*: 0**.009**
Type of cancer	Breast^a^	105.68	5684	2.25	0314	73.08	7461
	GIS^b^	107.03	5247	1.91	0316	69.97	9120
	Lung^c^	105.60	4418	2.18	0378	73.15	9566
	Hematologic^d^	109.17	4428	2.33	0119	71.83	7826
	Other^e^	106.56	5416	2.18	0611	74.85	11.006
		*t*: 1.172	***p***: 0.327**	*t*: 3.460 d > a > c = e > b	***p*: 0**.011**	*t*: 1.045	***p***: 0.388**
Time since cancer diagnosis	6 months or less^a^	108.08	4932	1.97	0430	75.52	9207
	7‐12 months^b^	107.56	5291	2.41	0639	76.31	9673
	13–18 months^c^	103.40	5902	2.27	0352	69.33	10.293
	19–24 months^d^	108.62	5501	2.25	0359	77.77	9993
	> 24 months^e^	105.89	4494	2.07	0293	69.14	6812
		*t*: 2.959 d > a > b > e > c	***p*: 0**.023**	*t*: 3.823 b > c > d > e > a	***p*: 0**.006**	*t*: 4.888 d > b > a > c > e	***p*: 0**.001**
Chemotherapy cycle number	3rd cycle	108.87	4865	1.99	0400	74.70	9281
	> 3 cycles	106.01	5140	2.18	0422	72.04	9216
		*t*: 0.426	***p*: 0**.018*	*t*: 0.033	***p***: 0.056*	*t*: 0.092	***p***: 0.221*
Other treatment type received	No other treatment	107.29	5984	2.16	0586	74.29	10.955
	Radiotherapy	106.81	5047	2.19	0383	72.38	8366
	Surgery	105.83	4701	2.08	0296	71.48	8730
		*t*: 0.750	***p***: 0.475**	*t*: 0.773	***p***: 0.464**	*t*: 0.822	***p***: 0.442**

*Note*: a,b,c,d,e.

*Independent samples *T*‐test,

**Oneway‐ ANOVA, Tamhane's T^2^.

This table presents the results of a multiple linear regression analysis assessing the effect of sociodemographic characteristics, disease characteristics, PRS, and N‐SAS scores on SCNS‐SF scores. According to the model, each additional chronic illness reduced the SCNS‐SF score by 3129 points, while each unit increase in the N‐SAS score increased the SCNS‐SF score by 6495 points. The model indicates that the number of chronic diseases and N‐SAS total score explain 27.1% of the variance in SCNS‐SF scores (*p* < 0.05).

The mediating role of PRS in the relationship between N‐SAS and SCNS‐SF scores was examined. N‐SAS had a statistically significant negative effect on PRS (*β* = −0.737, *p* = 0.018), explaining 5% of its variance (*R* ^2^ = 0.050). PRS did not have a statistically significant effect on SCNS‐SF (*β* = 0.017, *p* = 0.752). N‐SAS had a direct positive effect on SCNS‐SF (*β* = 0.012, *p* = 0.007), and a statistically significant indirect effect through PRS (*p* = 0.007), although the magnitude of the indirect effect was minimal (*β* = 0.000). Together, N‐SAS and PRS explained 6.4% of the total variance in SCNS‐SF scores (*R* ^2^ = 0.064). Table 3

**TABLE 3:** Determining the effect of research variables on SCNS‐SF scores using regression analysis.

Variables	*β*	SE	*β*	*t*	*p*	95% CI lower	95% CI upper
Age	−0.183	0.118	−0.216	−1.549	0.125	−0.418	0.052
Gender	−3.121	2.260	−0.169	−1.381	0.170	−7.605	1.363
Marital status	−0.701	5.489	−0.022	−0.128	0.899	−11.593	10.190
Educational status	1.802	1.117	0.178	1.612	0.110	−0.415	4019
Employment status	4962	2879	0.210	1.723	0.088	−0.751	10.674
Person living with	4.136	2.838	0.238	1.457	0.148	−1.495	9.767
Chronic illness (non‐cancer)	−3.129	1.249	−0.273	−2.505	**0.014**	−5.607	−0.651
Cancer type	0.039	0.578	0.008	0.068	0.946	−1.108	1.186
Time since diagnosis	−1.065	0.827	−0.187	−1.288	0.201	−2.705	0.576
Chemotherapy cycle	−2.724	3.249	−0.119	−0.838	0.404	−9.171	3.724
Other treatments	0.838	1.313	0.072	0.638	0.525	−1.768	3.444
PBL	−0.021	0.167	−0.012	−0.125	0.901	−0.353	0.311
N‐SCI	6.495	2.318	0.297	2.802	**0.006**	1.895	11.095

*Note*: Beta: Regression coefficient, *β*: standardized regression coefficient, *R* ^2^: Coefficient of determination.

Abbreviation: SE, standard error.

In this model, the effect of the PRS variable on the N‐SAS is mainly direct, positive and significant (*β* = −0.019; *p* = 0.011). However, it was determined that the effect of PRS on SCNS‐SF was not significant (*β* = 0.017; *p* = 0.752). Therefore, it is not possible to talk about an indirect effect of PRS via SCNS‐SF. The effects of N‐SAS and SCNS‐SF on each other are positive and significant (*β* = 0.012, *p* = 0.004).

## 4 Discussion

In this study, it was found that psychological resilience levels and symptom management among individuals undergoing chemotherapy varied according to variables such as educational status, time since diagnosis, and treatment duration. Additionally, supportive care needs were determined to differ based on educational level and treatment process. The findings obtained were discussed in light of the existing literature. Table 4

**TABLE 4:** Structural model analysis results (*n* = 113).

	PRS			N‐SAS		
	*β*	SE	*p*	*β*	SE	*p*
SCNS‐SF	0.017	0.053	0.752	0.012	0.004	**0.007**
*R* ^2^	0.001	—	—	0.064	—	—
PRS	—	—	—	−2.737	1.137	**0.018**
*R* ^2^	—	—	—	0.050	—	—
N‐SAS	2.737	1.137	**0.018**	—	—	—
*R* ^2^	0.050	—	—	—	—	—
SCNS‐SF	—	—	—	0.012	0.004	**0.004**
PRS	—	—	—	−0.019	0.007	**0.011**
*R* ^2^	—	—	—	0.118	—	—
Indirect effect	—	—	—	0.000	0.001	**0.007**

*Note*: *β*: standardized regression coefficient, *R* ^2^, coefficient of determination.

Abbreviation: SE, standard error.

Psychological resilience has been reported to enhance psychological well‐being in individuals undergoing chemotherapy (Baeda and Nurwahyuni 2022). However, it has also been suggested that the treatment process may reduce patients' psychological resilience (Xu et al. 2023). An individual's psychological resilience is influenced by multiple factors, one of which is educational level (Xu et al. 2023, Festerling et al. 2023). Individuals with higher educational attainment are better able to comprehend information regarding the illness and treatment process, giving them an advantage in developing problem‐solving and self‐management skills. This contributes positively to psychological resilience (Xu et al. 2023). The duration since cancer diagnosis may negatively affect psychological resilience. A study conducted on breast cancer patients found that psychological resilience scores were higher at the time of diagnosis but showed a significant decline after 1 year (Veličković et al. 2022). Similarly, it has been reported that resilience decreases over time in individuals diagnosed with colorectal cancer and those undergoing treatment for breast cancer (Veličković et al. 2022, Mohlin et al. 2021). Therefore, it is recommended that psychological resilience levels in cancer patients be regularly assessed and supported (Veličković et al. 2022, Mohlin et al. 2021, X. Wang et al. 2024). Psychological resilience is generally positively correlated with the level of education, with higher education levels facilitating better adaptation to illness (Janitra et al. 2024). In this study, the group with no literacy exhibited lower psychological resilience. Within this context, providing psychological support to patients with lower educational levels may facilitate their adaptation to the disease process.

Another factor affecting patients’ psychological resilience is the duration of treatment. In a study conducted with breast cancer patients, it was reported that psychological resilience reached its lowest level during the second cycle of treatment but fluctuated in subsequent cycles (Baeda and Nurwahyuni 2022). It has been emphasized that patients with high psychological resilience adapt better to the treatment process, and this adaptation further enhances resilience over time (Ulibarri‐Ochoa et al. 2024). The length of time since cancer diagnosis is positively associated with patients’ adaptation to the disease (Bury‐Kamińska 2023). However, it is known that this process is influenced by various individual, familial, and environmental factors that may affect adaptation in different ways (Park et al. 2022). These findings suggest that patients may be more vulnerable at the beginning of treatment, but as they adjust to the disease, their resilience can improve; however, prolonged treatment may lead to a subsequent decrease. Individuals with higher education levels are more inclined to conduct research to cope with the illness, which can be beneficial.

Studies indicate that cancer patients with low educational levels tend to have poorer quality of life and are more affected by symptoms such as pain, fatigue, and depression (Larsen et al. 2021, Naamala et al. 2024). Higher education can positively impact quality of life by enhancing symptom management, self‐efficacy, and coping skills (Xie et al. 2025). Although symptom burden is high in gastrointestinal cancers, symptom management in these patients reportedly becomes more effective over time (X. Wang et al. 2023, K. Wang et al. 2025). In cancer patients, quality of life related to symptoms significantly decreases during the active treatment phases (chemotherapy, radiotherapy, and the first months following surgery), but typically returns to pre‐treatment levels within 6–12 months after the completion of therapy. (Tesio et al. 2024). During the early stages of the disease, particularly within the first 24 months, the absence of symptom progression facilitates effective symptom management. Additionally, the experience accumulated during this period, in conjunction with the patient's educational level, may enhance coping skills development. This process positively impacts the patient's overall adaptation to the illness.

Supportive care needs among patients undergoing chemotherapy vary individually and according to disease‐specific factors. Research shows that individuals with low education levels and their families require more support in areas such as access to information, disease management, and psychosocial support (Attari et al. 2022). Conversely, individuals with higher education levels report less need for support or experience changes in the nature of their needs, as they have easier access to health information and better self‐management skills (Ullrich et al. 2021, Yeom and Lee 2022). Supportive care needs vary according to disease stage, treatment process, and individual factors; however, it has been reported that these needs increase as the disease duration lengthens (Attari et al. 2022, Hart et al. 2022). This indicates that longer disease duration generally leads to greater care and support requirements.

An increase in symptoms negatively affects psychological resilience in patients (Arefian et al. 2023). Conversely, as psychological resilience increases, symptom‐related quality of life improves (Yuan et al. 2024). During the chemotherapy process, enhancement of psychological resilience is closely associated with symptom alleviation and the improvement of patients’ quality of life (Ulibarri‐Ochoa et al. 2024). Improvements in symptom management (Li et al. 2023) and meeting patients’ supportive care needs facilitate adaptation to the illness (Hu et al. 2024). The literature indicates that psychological resilience has a significant effect on supportive care needs, with increased resilience leading to a reduction in these needs, thereby supporting the overall well‐being of the patient (Soyer Er and Erkan 2023). In this study, the effect of psychological resilience on supportive care needs was limited. This may be due to the fact that most participants in the study group were married and living with their spouses, potentially resulting in their supportive care needs being met by their partners.

## 5 Conclusion

Patients undergoing chemotherapy exhibit high levels of psychological resilience. They are not significantly affected by problems related to the disease or treatment. Individuals’ supportive care needs are at a moderate level. While an increase in symptoms negatively impacts psychological resilience, the effect of psychological resilience on supportive care needs is limited and indirect. Additionally, an increase in symptoms leads to greater supportive care needs among patients.

1. Recommendations for Nurses
2. Prioritize nursing interventions aimed at strengthening patients’ psychological resilience.
3. Develop holistic care plans that regularly assess symptom management and supportive care needs.
4. Provide integrated services combining psychological support, symptom control, and counseling.
5. Recommendations for Educators
6. Include more content on psychological resilience, symptom management, and supportive care in educational programs.
7. Guide students to assess patients’ psychosocial aspects and identify appropriate nursing approaches during clinical practice.
8. Support students in developing patient‐centered and holistic approaches, reinforced through case‐based learning methods.
9. Recommendations for Institutions
10. Strengthen specialized units such as psycho‐oncology support services and symptom management nursing.
11. Organize ongoing professional development programs for nurses in these areas.
12. Encourage multidisciplinary teamwork for supportive care services and develop individualized care plans based on patient needs.
13. Recommendations for Researchers
14. Conduct longitudinal studies exploring the relationships between psychological resilience, symptom management, and supportive care in greater depth.
15. Perform comparative studies with similar scales across different patient groups.
16. Test the effectiveness of nursing interventions aimed at enhancing psychological resilience through intervention‐based research.

### 5.1 Limitations of the Study

This study was conducted exclusively with patients from the chemotherapy unit of a single institution, which may limit the generalizability of the results. Furthermore, some patients perceived the scale items as excessive and chose to temporarily discontinue participation, which may have affected the consistency of data collection.

## Author Contributions

**Rukiye Burucu**:: methodology, formal analysis, supervision. **Işın Cantekin**: data curation, writing–original draft, resources, supervision.

## Data Availability

The data that support the findings of this study are available on request from the corresponding author. The data are not publicly available due to privacy or ethical restrictions.
